# Complete Genome Sequence of Legionella cardiaca Strain H63^T^, Isolated from a Case of Native Valve Endocarditis

**DOI:** 10.1128/mra.00175-23

**Published:** 2023-06-13

**Authors:** Alberto E. Lopez, Joshua Mayoral, Nicholas P. Cianciotto

**Affiliations:** a Department of Microbiology and Immunology, Northwestern University Medical School, Chicago, Illinois, USA; Wellesley College

## Abstract

We report the complete genome sequence of Legionella cardiaca strain H63^T^, which had been isolated from aortic valve tissue from a patient with native endocarditis. The genome assembly contains a single 3,477,232-bp contig, with a G+C content of 38.59%, and is predicted to encode 2,948 proteins.

## ANNOUNCEMENT

Among the extrapulmonary manifestations of *Legionella* infection is endocarditis (1, 2). Our laboratory previously described a novel isolate that had been obtained by plating material from resected aortic valve tissue on buffered charcoal yeast extract (BCYE) agar at 37°C and was named Legionella cardiaca strain H63^T^ (ATCC BAA-2315) (3, 4). Because prior genotypic analysis of L. cardiaca involved only DNA-DNA hybridization and phylogenetic analyses of three loci (4) and the mechanisms of *Legionella* endocarditis are unknown, we determined the complete genome of L. cardiaca H63^T^.

Using the Promega Maxwell 16 system, DNA was isolated from H63^T^, which had been grown from a single colony to confluence on BCYE agar at 37°C for 3 days. DNA was sequenced using Illumina and Pacific Biosciences (PacBio) platforms. For Illumina sequencing, short-read libraries were generated with a KAPA HyperPrep kit (Roche) and sequenced using 150-bp paired-end reads on a NovaSeq 6000 system. For PacBio sequencing, genomic DNA (gDNA) was fragmented to an average size of ~11 kb with a Covaris g-TUBE. DNA was cleaned with SPRIselect beads, followed by library construction using the SMRTbell Express template preparation kit v2.0 (PacBio), which includes single-strand DNA overhang removal, DNA damage repair, end repair/A-tailing, and barcoded overhang adaptor ligation. The library was pooled with other libraries on an equimolar basis and subsequently size selected on a BluePippin instrument with an 8-kb cutoff value. The library pool was purified with SPRIselect beads, quantified with a Qubit 4.0 fluorometer, and assessed with an Agilent fragment analyzer. The final library pool was sequenced with PacBio Sequel II v2.0 chemistry and a single-molecule real-time (SMRT) Cell 8M on a Sequel II instrument at an on-plate concentration of 85 pM. Illumina reads were quality filtered using a combination of Illumina RTA v1.8.70.0 and Trimmomatic v0.38.0 (5). PacBio reads were quality filtered using FastQC v0.72 (6). For Illumina sequencing, 5,802,382 reads were generated, with ~250× coverage; for PacBio sequencing, 1,609,389 reads (*N*_50_, 11,074 bp) were generated. The resulting raw sequencing reads were processed using PacBio SMRTLink v9.0, including demultiplexing by Lima v1.11.0 (7). Genome assembly was performed using the PacBio HGAP4 assembler, which includes overlap determination, followed by consensus polishing with Pilon v1.24 (8) using Illumina 150-bp paired-end reads generated from the same gDNA. Rotation of the chromosome was performed using the IGS automated prokaryotic annotation pipeline (9). The assembly yielded a single closed circular chromosome. Gene annotation was performed using the NCBI Prokaryotic Genome Annotation Pipeline (PGAP) v6.4 (10–12).

**FIG 1:**
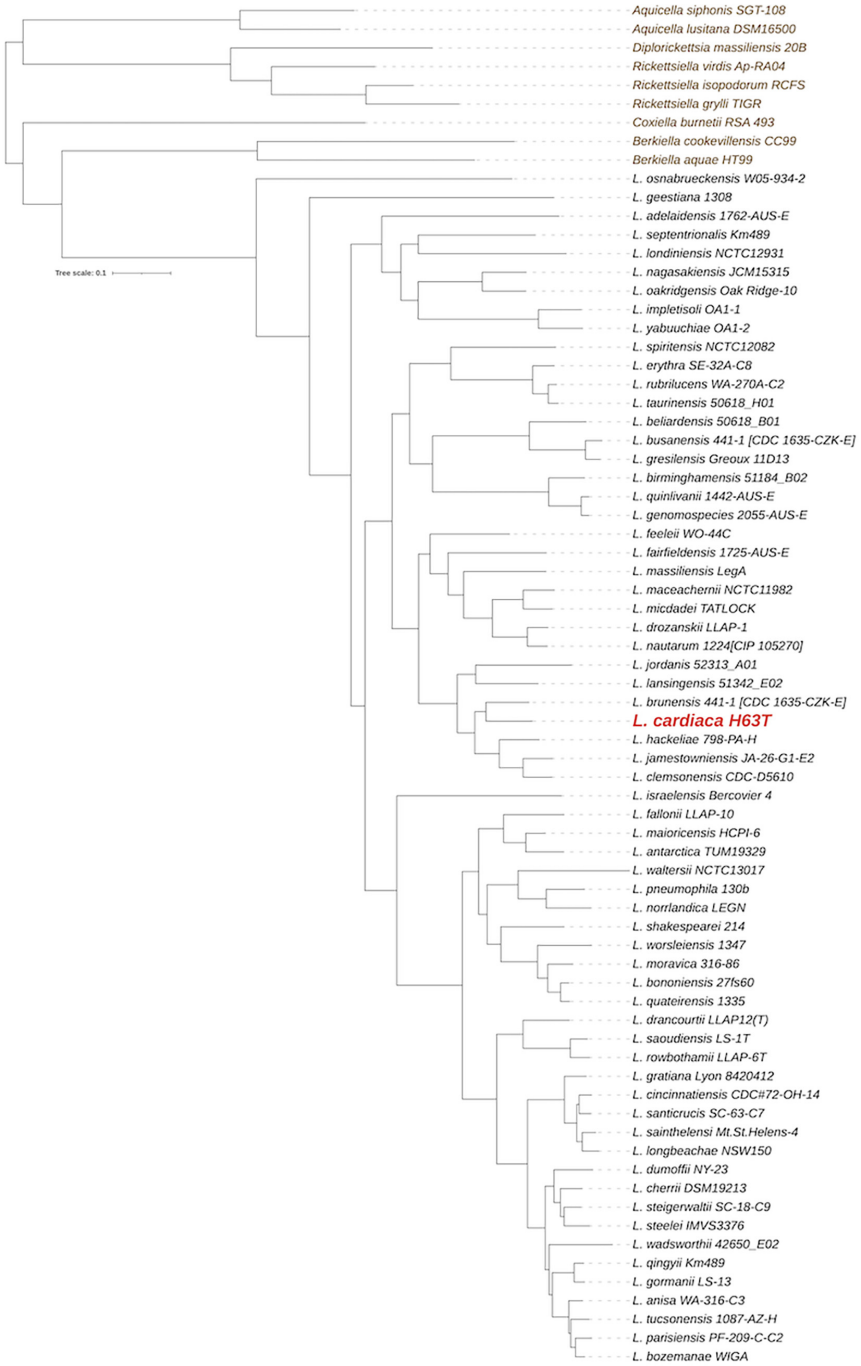
Relationships of L. cardiaca H63^T^ with 63 other sequenced species of *Legionella*. In the rooted species tree, L. cardiaca is highlighted in red and the other *Legionella* species and their corresponding strain names appear in black. Appearing at the top of the tree are non-*Legionella* species (in brown) that belong to other genera within the order *Legionellales*. Bar, 0.1 amino acid substitutions per site.

The H63^T^ genome assembly contains a single 3,477,232-bp contig (~4,411× coverage), with a G+C content of 38.593%, and is predicted to encode 2,948 proteins. A rooted species tree based on the concatenated amino acid alignment of 219 single-copy orthologous proteins was generated using OrthoFinder v2.5.4 (13–16), and strain H63^T^ was most closely related to Legionella brunensis (Fig. 1). Pairwise average nucleotide identity (ANI) comparisons (17–21) confirmed that L. cardiaca is a distinct species within the L. brunensis-containing clade (Table 1), which is linked to disease (4, 22–29). Consistent with H63^T^ being virulent in infection models (4), its genome has genes encoding a type IVB secretion system and a type II secretion system and genes linked to iron assimilation (30–38).

**TABLE 1:** ANI values from pairwise comparisons between the genome of L. cardiaca strain H63^T^ and the genomes of the *Legionella* species most closely related to strain H63^T^

Subject strain for query	GenBank assembly accession no.	ANI (%)
Legionella brunensis	GCF_001467025.1	76.56
Legionella hackeliae	GCF_001467705.1	74.96
Legionella clemsonensis	GCF_002240035.1	74.93
Legionella jamestowniensis	GCF_900640205.1	74.39
Legionella lansingensis	GCF_000622185.1	74.22
Legionella jordanis	GCF_001467765.1	71.95
Legionella feeleii	GCF_001467625.1	72.46
Legionella fairfieldensis	GCF_000621525.1	72.05
Legionella drozanskii	GCF_001467585.1	71.81
Legionella nautarum	GCF_001467895.1	71.77
Legionella maceachernii	GCF_001467845.1	71.55
Legionella micdadei	GCF_001467875.1	71.36
Legionella massiliensis	GCF_000756815.1	71.09

### Data availability.

The assembly of the genome is available under GenBank accession number CP119078, and raw reads have been submitted to the NCBI SRA under accession numbers SRR23636844 and SRR23636845.
